# 3-(2-Fluoro­phen­oxy)propanoic acid

**DOI:** 10.1107/S1600536810049974

**Published:** 2010-12-15

**Authors:** Yao-Yuan Zhou, Xiao-Bo Gu, Meng-Jun Jiang, Gang-Ming Cai

**Affiliations:** a Key Laboratory of Nuclear Medicine, Ministry of Health, Jiangsu Key Laboratory of Molecular Nuclear Medicine, Jiangsu Institute of Nuclear Medicine, Wuxi 214063, People’s Republic of China

## Abstract

In the title compound, C_9_H_9_FO_3_, the dihedral angle between the carboxyl group and the benzene ring is 79.4 (3)°. In the crystal, mol­ecules form centrosymmetric dimers through pairs of classical O—H⋯O hydrogen bonds. These are further linked by weaker C—H⋯O inter­actions, forming a three-dimensional network.

## Related literature

For a related structure, see: Potrzebowski & Chruszcz (2007 ▶). For the synthesis, see: Bäurle *et al.* (2006 ▶).
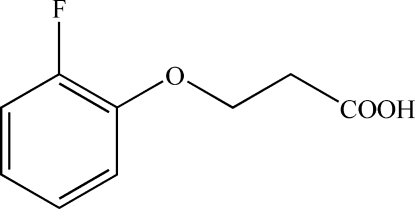


## Experimental

### 

#### Crystal data

C_9_H_9_FO_3_
*M* *_r_* = 184.16
Monoclinic, 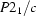

*a* = 13.934 (16) Å
*b* = 4.974 (5) Å
*c* = 13.098 (14) Å
β = 110.546 (12)°
*V* = 850.0 (16) Å^3^
*Z* = 4
Mo *K*α radiation
μ = 0.12 mm^−1^
*T* = 153 K
0.45 × 0.30 × 0.08 mm

#### Data collection

Rigaku AFC10/Saturn724+ diffractometer
Absorption correction: multi-scan (*CrystalClear*; Rigaku, 2008 ▶) *T*_min_ = 0.947, *T*_max_ = 0.990
5881 measured reflections
1518 independent reflections
1034 reflections with *I* > 2σ(*I*)
*R*_int_ = 0.070

#### Refinement

*R*[*F* ^2^ > 2σ(*F* ^2^)] = 0.080
*wR*(*F* ^2^) = 0.189
*S* = 0.98
1518 reflections
122 parameters
H atoms treated by a mixture of independent and constrained refinement
Δρ_max_ = 0.42 e Å^−3^
Δρ_min_ = −0.38 e Å^−3^

### 

Data collection: *CrystalClear* (Rigaku, 2008 ▶); cell refinement: *CrystalClear*; data reduction: *CrystalClear*; program(s) used to solve structure: *SHELXS97* (Sheldrick, 2008 ▶); program(s) used to refine structure: *SHELXL97* (Sheldrick, 2008 ▶); molecular graphics: *SHELXTL* (Sheldrick, 2008 ▶); software used to prepare material for publication: *SHELXTL*.

## Supplementary Material

Crystal structure: contains datablocks I, global. DOI: 10.1107/S1600536810049974/sj5066sup1.cif

Structure factors: contains datablocks I. DOI: 10.1107/S1600536810049974/sj5066Isup2.hkl

Additional supplementary materials:  crystallographic information; 3D view; checkCIF report

## Figures and Tables

**Table 1:** Hydrogen-bond geometry (Å, °)

*D*—H⋯*A*	*D*—H	H⋯*A*	*D*⋯*A*	*D*—H⋯*A*
O3—H4*O*⋯O2^i^	0.91 (7)	1.77 (7)	2.671 (6)	177 (7)
C4—H4⋯O1	0.95	2.57	3.519 (7)	176

Symmetry code: (i) 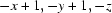.

**Fig. 1.**
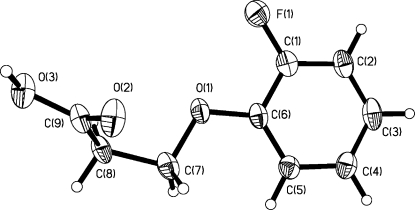
A view of the title compound with the atomic numbering scheme. Displacement ellipsoids are drawn at the 50% probability level.

## supplementary crystallographic information

### Comment

The title compound,(I), is an important intermediate in the synthesis of 8-fluorochroman-4-one (Bäurle *et al.*, 2006). We report herein its structure (Fig.1).

The bond lengths and angles in (I) are within normal ranges (Potrzebowski & Chruszcz, 2007). The dihedral angle between the C1—C6 benzene ring and the C9/O2/O3 plane is 79.4 (3) °. In the crystal, molecules form centrosymmetric dimers through classical O3—H4O···O2 hydrogen bonds (Table 1). These are further linked by weaker C4—H4···O1 contacts forming a three-dimensional network.

### Experimental

The title compound was crystallized from dichloromethane and hexane (1:1); colorless block-shaped crystals were obtained after several days.

### Refinement

The crystals were not of good quality resulting in uncertainties in unit cell dimensions and other metrical data being somewhat higher than normal. Positional parameters of all the H atoms bonded to C atoms were calculated geometrically and were allowed to ride on the C atoms to which they were bonded, with C—H distances of 0.95Å (CH), 0.99Å (CH_2_), and with *U*iso(H) =1.2*U*eq of the parent atoms. The H-atom of the OH group was located in a difference map and allowed to refine freely with an isotropic displacement parameter.

### Crystal data

**Table:**

C_9_H_9_FO_3_	*F*(000) = 384
*M**_r_* = 184.16	*D*_x_ = 1.439 Mg m^−^^3^
Monoclinic, *P*2_1_/*c*	Mo *K*α radiation, λ = 0.71073 Å
Hall symbol: -P 2ybc	Cell parameters from 2182 reflections
*a* = 13.934 (16) Å	θ = 3.1–27.5°
*b* = 4.974 (5) Å	µ = 0.12 mm^−^^1^
*c* = 13.098 (14) Å	*T* = 153 K
β = 110.546 (12)°	Block, colorless
*V* = 850.0 (16) Å^3^	0.45 × 0.30 × 0.08 mm
*Z* = 4	

### Data collection

**Table:**

Rigaku AFC10/Saturn724+ diffractometer	1518 independent reflections
Radiation source: Rotating Anode	1034 reflections with *I* > 2σ(*I*)
graphite	*R*_int_ = 0.070
Detector resolution: 28.5714 pixels mm^-1^	θ_max_ = 25.5°, θ_min_ = 3.1°
φ and ω scans	*h* = −16→16
Absorption correction: multi-scan (*CrystalClear*; Rigaku, 2008)	*k* = −6→6
*T*_min_ = 0.947, *T*_max_ = 0.990	*l* = −15→15
5881 measured reflections	

### Refinement

**Table:**

Refinement on *F*^2^	Primary atom site location: structure-invariant direct methods
Least-squares matrix: full	Secondary atom site location: difference Fourier map
*R*[*F*^2^ > 2σ(*F*^2^)] = 0.080	Hydrogen site location: inferred from neighbouring sites
*wR*(*F*^2^) = 0.189	H atoms treated by a mixture of independent and constrained refinement
*S* = 0.98	*w* = 1/[σ^2^(*F*_o_^2^) + (0.0106*P*)^2^ + 5.690*P*] where *P* = (*F*_o_^2^ + 2*F*_c_^2^)/3
1518 reflections	(Δ/σ)_max_ < 0.001
122 parameters	Δρ_max_ = 0.42 e Å^−^^3^
0 restraints	Δρ_min_ = −0.38 e Å^−^^3^

### Special details

**Table:**

Geometry. All e.s.d.'s (except the e.s.d. in the dihedral angle between two l.s. planes) are estimated using the full covariance matrix. The cell e.s.d.'s are taken into account individually in the estimation of e.s.d.'s in distances, angles and torsion angles; correlations between e.s.d.'s in cell parameters are only used when they are defined by crystal symmetry. An approximate (isotropic) treatment of cell e.s.d.'s is used for estimating e.s.d.'s involving l.s. planes.
Refinement. Refinement of *F*^2^ against ALL reflections. The weighted *R*-factor *wR* and goodness of fit *S* are based on *F*^2^, conventional *R*-factors *R* are based on *F*, with *F* set to zero for negative *F*^2^. The threshold expression of *F*^2^ > σ(*F*^2^) is used only for calculating *R*-factors(gt) *etc*. and is not relevant to the choice of reflections for refinement. *R*-factors based on *F*^2^ are statistically about twice as large as those based on *F*, and *R*- factors based on ALL data will be even larger.

### Fractional atomic coordinates and isotropic or equivalent isotropic displacement parameters (Å^2^)

**Table:**

	*x*	*y*	*z*	*U*_iso_*/*U*_eq_	
F1	0.1148 (2)	0.0797 (6)	0.0408 (2)	0.0401 (7)	
O1	0.2551 (2)	0.4195 (6)	0.1573 (2)	0.0279 (7)	
O2	0.4656 (2)	0.4357 (6)	0.1091 (3)	0.0358 (8)	
O3	0.4220 (2)	0.7887 (6)	−0.0035 (3)	0.0350 (8)	
C8	0.3591 (3)	0.7823 (8)	0.1414 (4)	0.0279 (10)	
H8A	0.2946	0.8508	0.0873	0.034*	
H8B	0.3981	0.9392	0.1815	0.034*	
C6	0.2124 (3)	0.2528 (8)	0.2131 (4)	0.0239 (9)	
C5	0.2368 (3)	0.2465 (8)	0.3253 (3)	0.0264 (9)	
H5	0.2869	0.3663	0.3705	0.032*	
C1	0.1372 (3)	0.0743 (9)	0.1491 (4)	0.0281 (10)	
C3	0.1149 (3)	−0.1099 (9)	0.3076 (4)	0.0339 (11)	
H3	0.0820	−0.2334	0.3399	0.041*	
C9	0.4201 (3)	0.6507 (8)	0.0816 (4)	0.0279 (10)	
C7	0.3327 (3)	0.6037 (8)	0.2214 (4)	0.0288 (10)	
H7A	0.3941	0.5042	0.2677	0.035*	
H7B	0.3064	0.7129	0.2691	0.035*	
C4	0.1878 (3)	0.0641 (9)	0.3721 (4)	0.0301 (10)	
H4	0.2050	0.0603	0.4490	0.036*	
C2	0.0898 (3)	−0.1050 (9)	0.1967 (4)	0.0304 (10)	
H2	0.0395	−0.2255	0.1523	0.036*	
H4O	0.462 (5)	0.712 (14)	−0.037 (5)	0.08 (2)*	

### Atomic displacement parameters (Å^2^)

**Table:**

	*U*^11^	*U*^22^	*U*^33^	*U*^12^	*U*^13^	*U*^23^
F1	0.0365 (15)	0.0424 (16)	0.0402 (16)	−0.0105 (12)	0.0121 (12)	0.0004 (13)
O1	0.0313 (15)	0.0225 (15)	0.0344 (17)	−0.0050 (12)	0.0170 (13)	0.0010 (13)
O2	0.0391 (18)	0.0272 (16)	0.052 (2)	0.0124 (14)	0.0289 (16)	0.0122 (15)
O3	0.0405 (18)	0.0250 (16)	0.050 (2)	0.0084 (14)	0.0291 (16)	0.0076 (15)
C8	0.029 (2)	0.0163 (19)	0.044 (3)	−0.0018 (17)	0.0197 (19)	−0.0018 (18)
C6	0.024 (2)	0.0166 (19)	0.036 (2)	0.0033 (15)	0.0168 (17)	0.0019 (17)
C5	0.032 (2)	0.019 (2)	0.030 (2)	0.0035 (16)	0.0134 (18)	−0.0005 (17)
C1	0.024 (2)	0.025 (2)	0.037 (3)	0.0029 (17)	0.0131 (18)	0.0024 (19)
C3	0.033 (2)	0.025 (2)	0.057 (3)	0.0042 (18)	0.031 (2)	0.009 (2)
C9	0.027 (2)	0.020 (2)	0.041 (3)	−0.0025 (17)	0.0175 (19)	0.0010 (19)
C7	0.034 (2)	0.017 (2)	0.043 (3)	0.0003 (17)	0.023 (2)	−0.0049 (19)
C4	0.033 (2)	0.026 (2)	0.035 (2)	0.0088 (18)	0.0168 (19)	0.0066 (19)
C2	0.022 (2)	0.025 (2)	0.045 (3)	0.0006 (17)	0.0131 (19)	0.006 (2)

### Geometric parameters (Å, °)

**Table:**

F1—C1	1.342 (5)	C6—C1	1.405 (6)
O1—C6	1.372 (5)	C5—C4	1.399 (6)
O1—C7	1.441 (5)	C5—H5	0.9500
O2—C9	1.231 (5)	C1—C2	1.381 (6)
O3—C9	1.317 (5)	C3—C2	1.370 (7)
O3—H4O	0.90 (7)	C3—C4	1.376 (7)
C8—C9	1.494 (6)	C3—H3	0.9500
C8—C7	1.515 (6)	C7—H7A	0.9900
C8—H8A	0.9900	C7—H7B	0.9900
C8—H8B	0.9900	C4—H4	0.9500
C6—C5	1.387 (6)	C2—H2	0.9500
			
C6—O1—C7	116.8 (3)	C2—C3—H3	120.0
C9—O3—H4O	113 (4)	C4—C3—H3	120.0
C9—C8—C7	115.3 (3)	O2—C9—O3	122.7 (4)
C9—C8—H8A	108.4	O2—C9—C8	123.8 (4)
C7—C8—H8A	108.4	O3—C9—C8	113.5 (4)
C9—C8—H8B	108.4	O1—C7—C8	106.5 (4)
C7—C8—H8B	108.4	O1—C7—H7A	110.4
H8A—C8—H8B	107.5	C8—C7—H7A	110.4
O1—C6—C5	126.0 (4)	O1—C7—H7B	110.4
O1—C6—C1	115.9 (4)	C8—C7—H7B	110.4
C5—C6—C1	118.2 (4)	H7A—C7—H7B	108.6
C6—C5—C4	120.2 (4)	C3—C4—C5	120.5 (4)
C6—C5—H5	119.9	C3—C4—H4	119.8
C4—C5—H5	119.9	C5—C4—H4	119.8
F1—C1—C2	121.3 (4)	C3—C2—C1	120.3 (4)
F1—C1—C6	117.8 (4)	C3—C2—H2	119.8
C2—C1—C6	120.9 (4)	C1—C2—H2	119.8
C2—C3—C4	119.9 (4)		
			
C7—O1—C6—C5	0.3 (6)	C7—C8—C9—O3	165.5 (4)
C7—O1—C6—C1	−179.8 (3)	C6—O1—C7—C8	−174.2 (3)
O1—C6—C5—C4	−179.4 (4)	C9—C8—C7—O1	−72.7 (5)
C1—C6—C5—C4	0.7 (6)	C2—C3—C4—C5	−0.1 (6)
O1—C6—C1—F1	0.8 (5)	C6—C5—C4—C3	−0.2 (6)
C5—C6—C1—F1	−179.3 (3)	C4—C3—C2—C1	−0.1 (6)
O1—C6—C1—C2	179.2 (4)	F1—C1—C2—C3	179.0 (4)
C5—C6—C1—C2	−0.9 (6)	C6—C1—C2—C3	0.6 (6)
C7—C8—C9—O2	−15.4 (6)		

### Hydrogen-bond geometry (Å, °)

**Table:**

*D*—H···*A*	*D*—H	H···*A*	*D*···*A*	*D*—H···*A*
O3—H4O···O2^i^	0.91 (7)	1.77 (7)	2.671 (6)	177 (7)
C4—H4···O1^ii^	0.95	2.57	3.519 (7)	176

Symmetry codes: (i) −*x*+1, −*y*+1, −*z*; (ii) .

## References

[bb1] Bäurle, S., Berger, M. & Jaroch, S. (2006). WO Patent 2006/027236.

[bb2] Potrzebowski, W. & Chruszcz, M. (2007). *Acta Cryst.* E**63**, o2754.

[bb3] Rigaku (2008). *CrystalClear* Rigaku Corporation, Tokyo, Japan.

[bb4] Sheldrick, G. M. (2008). *Acta Cryst.* A**64**, 112–122. 10.1107/S0108767307043930 18156677

